# Evaluation of a Prednisolone Acetate-Loaded Subconjunctival Implant for the Treatment of Recurrent Uveitis in a Rabbit Model

**DOI:** 10.1371/journal.pone.0097555

**Published:** 2014-05-19

**Authors:** Marcus Ang, Xuwen Ng, Cheewai Wong, Peng Yan, Soon-Phaik Chee, Subbu S. Venkatraman, Tina T. Wong

**Affiliations:** 1 Singapore National Eye Centre, Singapore, Singapore; 2 Singapore Eye Research Institute, Singapore, Singapore; 3 Materials Science and Engineering, Nanyang Technological University, Singapore, Singapore; Oregon Health & Science University, United States of America

## Abstract

**Aim:**

To assess the efficacy of a biodegradable, prednisolone acetate implant in a rabbit uveitis model.

**Methods:**

Randomized, controlled study of biodegradable microfilms preloaded with prednisolone acetate (PA) in a rabbit uveitis model. Experimental uveitis was induced by unilateral intravitreal injection of *Mycobacterium tuberculosis* H37Ra antigen (50 ug; 1 ug/uL) in preimmunized rabbits. PA-loaded poly[d,l-lactide-co-ε-caprolactone] (PLC) microfilms (n = 10) and blank microfilms (n = 6) were implanted subconjunctivally. An estimate of PA release *in vivo* was calculated from measured residual PA amounts in microfilms after the rabbits were sacrificed. The eyes were clinically monitored for ocular inflammation for 28 days. Histopathological examination of the enucleated eyes was performed at the end of the study period.

**Results:**

*In vitro* studies revealed that sandwich PA-loaded microfilm formulations exhibited higher release kinetic compared to homogenous PA-loaded microfilms. The 60–40–60% microfilm released an average of 0.034 mg/day of PA over the period of 60 days *in vitro*; and we found that approximately 0.12 mg/day PA was released *in vivo*. Animals implanted with the PA-loaded microfilms exhibited significantly lowered median inflammatory scores when compared against the control group in this model for recurrent uveitis (P<0.001). The implants were clinically well tolerated by all the animals. Histology results showed no significant scarring or inflammation around the PA-loaded microfilms.

**Conclusion:**

Our pilot study demonstrated that a subconjunctival PA-loaded implant is effective in suppressing inflammation in the rabbit model of uveitis, by providing therapeutic levels of PA that attenuated the inflammatory response even after a rechallenge. Longer term studies are now needed to establish the therapeutic potential of such a delivery system for treatment of ocular inflammation.

## Introduction

Uveitis is an inflammatory disorder affecting the iris, ciliary body or choroid, which is a relatively common eye disorder, with an estimated incidence rate of 17 and 22.6 per 100,000 person years[1], [2]. Inadequate diagnosis and treatment in severe or prolonged ocular inflammation may lead to sight threatening complications [1]. Currently, the mainstay of treatment is corticosteroids, which may be administered topically or as a periocular/intravitreal injection, with or without the concurrent use of oral steroids [2]. There are, however, problems associated with these routes of administration. Topical steroids have poor ocular penetration and rapid clearance from the eye necessitating frequent application. Patients who require a higher intraocular concentration of steroid than topical steroids can provide may be given periocular injections - but the drug is rapidly cleared within 2 weeks of administration[3] and frequent injections are associated with risks of globe perforation and retrobulbar haemorrhage. In patients with posterior uveitis, topical steroids are often unable to control inflammation due to poor ocular penetration to the posterior segment. In these patients, intravitreal administration delivers the highest concentration of steroid and sustained release intravitreal implants can provide therapeutic drug levels for up to 6 months[4]. The disadvantages of this route of administration are the increased risk of raised intraocular pressure, endophthalmitis and retinal detachment. Moreover, should these corticosteroid-related complications such as raised intraocular pressure leading to glaucoma occur, removal of the steroid implant from the posterior segment would be difficult.

A subconjunctival implant may circumvent the risks involved with intravitreal administration; and due to its anatomical siting, may be removed relatively easily if necessary. Prednisolone acetate achieves the highest aqueous concentration within 2 hours and maintains higher levels for 24 hours, compared to dexamethasone and other commonly used corticosteroids[5]. We have demonstrated in previous studies the anti-fibrotic and anti-inflammatory properties of a subconjunctivally implanted prednisolone acetate (PA)-preloaded microfilm in the rabbit model of subconjunctival scarring following glaucoma filtration surgery [6] and rat keratoplasty model[7]. Our *in vivo* studies have shown that PA-loaded poly[d,l-lactide-co-ε-caprolactone] (PLC) microfilms display good biocompatibility, feasibility, and desirable sustained drug release profiles, maintaining high anterior chamber PA levels at 76.7±5.9, 70.3±2.3, and 42.7±4.1 ng/mL at 2, 4, and 12 weeks, respectively[8], [9].

Thus, in this study, we sought to determine whether the biodegradable PA-loaded microfilm is able to deliver sustained therapeutic levels of corticosteroid to effectively reduce ocular inflammation and attenuate the intensity of recurrence uveitis following a rechallenge in the rabbit model of uveitis.

## Methods

### In Vitro Study

**Figure 1:**
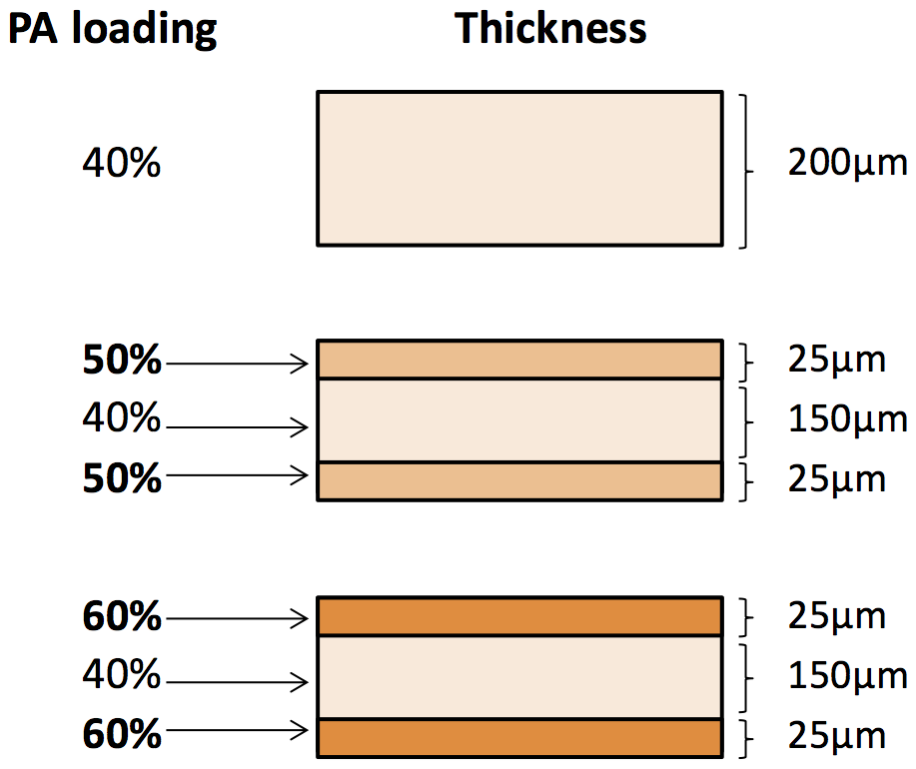
Figure depicting the single layer and tri-layer matrices with their respective drug loading and thickness.

Polymeric films were prepared using a previously described solution casting method, using biomedical grade of copolymer poly(d-, l-lactide-co-ε-caprolactone) or PLC70/30 (l-lactide to ε-caprolactone molar ratio = 70/30, with intrinsic viscosity of 1.6 dl/g) (Purac Far East, Singapore) and prednisolone 21-acetate (≥97%) (Sigma-Aldrich, Singapore)[10]. High performance liquid chromatography (HPLC) grade dichloromethane (DCM) and acetone nitrile (ACN) were used as received. Phosphate buffer saline pellets were purchased from Sigma-Aldrich and prepared in accordance to the manufacturer's protocol. PLC70/30 with predetermined PA drug-loading percentage of 40, 50 and 60 wt % were dissolved in DCM to form a polymer-drug solution - **Table 1** **.** For the single layer film formulation, the films were prepared by solution casting a single drug-polymer solution on the glass plate using an automatic film applicator. For the tri-layer film formulations, drug layers were cast layer by layer with a 10 minutes interval between each cast. **Figure 1** depicts the respective drug loading and thickness of each film.

**Table 1:** Various microfilm formulations analyzed during *in vitro* study.

Formulation combination	Type	First Layer	Second Layer	Third Layer
40%	Single layer	PLC with 40 wt% PA; 200 µm	Nil	Nil
50–40–50%	Sandwich	PLC with 50 wt% PA; 25 µm	PLC with 40 wt% PA; 150 µm	PLC with 50 wt% PA; 25 µm
60–40–60%	Sandwich	PLC with 60 wt% PA; 25 µm	PLC with 40 wt% PA; 150 µm	PLC with 60 wt% PA; 25 µm
PLC = poly[d,l-lactide-co-ε-caprolactone]; PA = prednisolone acetate; wt% = weight percentage				

Subsequently, all the films were dried in room-temperature ambience for 1 day, followed by drying in the 37°C vacuum oven for 1 week. The residual solvent was measured using a thermogravimetric analyzer (TGA, TA instruments Q500) and verified to be less than 1% before use. After drying, all samples were cut to the desired dimensions (4.0×8.0×0.2 mm) with their edges rounded for *in vitro* and *in vivo* studies. All the samples were sterilized by room temperature-ethylene oxide (RT-ETO) prior *in vivo* implantation. For the *in vitro* drug release study, three PA-loaded microfilms of the single and tri-layered formulations were studied *in vitro* to determine the rate of drug release. All samples were immersed in PBS in the 37°C incubator throughout the study and the amount of PA released in PBS over time was quantified using the HPLC. The PA-loaded microfilm with the optimal PA release was selected for the *in vivo* study.

### In Vivo Study

**Figure 2:**
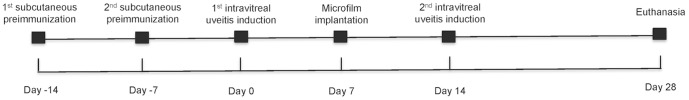
Flow chart to describe our study design. Days -14 to Day 0 is the preimmunization phase. The first intravitreal uveitis induction was performed on Day 0. The 2^nd^ intravitreal uveitis induction on Day 14 simulates a recurrence of uveitis.

This is a double armed, parallel designed, randomized, placebo controlled study to assess the efficacy of a subconjunctivally implanted biodegradable PA-loaded microfilm in attenuating the inflammation in an animal model of experimental uveitis. **Figure 2** shows a summary of our study design.

### Animals

Approval was obtained from the SingHealth Institute Animal Care and Use Committee (IACUC Singhealth Approval Number 2012/SHS/730) and all procedures were performed in accordance with the ARVO Statement for the Use of Animals in Ophthalmic and Vision Research. 16 Adult male New Zealand White rabbits, weighing 2-2.5 kg were used in this study. All rabbits were examined with a slit lamp and only rabbits with no ocular pathology were included in the study. In the placebo arm of the study, rabbits received subconjunctival implantation of a blank microfilm containing no PA. In the treatment arm, rabbits received subconjunctival implantation of the PA-loaded microfilm. Six rabbits were randomized into the placebo arm and 10 into the treatment arm.

### Induction of experimental uveitis

A subcutaneous injection of *Mycobacterium tuberculosis* H37Ra antigen (10 mg; Difco, Detroit, MI) suspended in mineral oil (500 uL) was given as preimmunization.[11] Successful preimmunization was confirmed after one week by the presence of a visible skin nodule at the injection site. Uveitis was induced on Days 0 and 14 of the study – **Figure 2**. The rabbits were anesthetized with intraperitoneal injection of ketamine hydrochloride (5 mg/kg) and xylazine hydrochloride (2 mg/kg). Following topical anaesthesia (Minims Tetracaine Hydrochloride 0.5%; Bausch and Lomb, UK) the right eye of each rabbit was disinfected with 5% povidone iodine. An intravitreal injection of *Mycobacterium tuberculosis* H37Ra antigen suspended in sterile saline (50 ug; 1 ug/uL) using a Hamilton syringe with a 31-gauge needle was given through the superotemporal sclera, 1.5 mm from the limbus. One drop of tobramycin 0.3% ophthalmic solution (Alcon Lab. USA) was instilled at the end of the procedure.

### Implantation of microfilms

Implantation of microfilms was performed on Day 7 i.e. 7 days after the first uveitis intravitreal induction, by 2 masked independent investigators (MA, CW). Each rabbit was anesthetized with intraperitoneal injection of ketamine hydrochloride (5 mg/kg) and xylazine hydrochloride (2 mg/kg). After the animal had been adequately anaesthetized, the eye was cleaned with povidone–iodine (10%) and draped with sterile cloth. A subconjunctival pocket was created via blunt dissection just at the limbus with a 5–6 mm incision in the superior-temporal aspect of the eye. Microfilms were sterilized in ethyl alcohol and chlorhexidine before soaking in sterile normal saline. The microfilm was then inserted into the subconjunctival pocket 1 mm from the limbus using a conjunctival forceps. Closure with 10-0 nylon sutures was performed to secure implantation of each microfilm to the sclera. Topical tobramycin 0.3% ophthalmic solution (Alcon Lab. USA) was administered in each eye 4 times a day for 5 days.

### Clinical Examination

Daily visual inspection of the operated eyes following surgery was conducted to document any changes at the implant site, gross appearance of the microfilm implants and for evidence of local erosion of the implant or infection by 1 masked independent investigator (CW). Slit-lamp biomicroscopy, photography of the anterior segment and dilated fundal examination with binocular indirect ophthalmoscopy using a 20 D lens was performed prior to uveitis induction and at 8 defined time points thereafter (Days 1, 4, 8, 13, 15, 19, 22 and 28). Clinical severity of uveitis was scored using anterior chamber cells/flares, vitreous haze, and iris vessels as described in previous literature[12], [13].

### Enucleation, euthanasia & pathology procedures

All rabbits were euthanized at the end of the study period of 28 days. Euthanasia was carried out with intraperitoneal pentobarbitone (60–150 mg/kg) followed by enucleation and immersing the eyes in a mixture of 4% paraformaldehyde and 2.5% neutral buffered formalin for 24 hours. The globes were dehydrated, embedded in paraffin and sent for microtome sectioning and staining (Sirius red F3BA; Sigma, St. Louis, MO). We used polarization microscopy of stained collagen fibers to reveal gross collagen bundling patterns to assess fibrosis and scarring. Next, sectioned slides were heated, deparaffinized, and rehydrated before antigen retrieval, by incubating in citrate buffer at 95–100°C. Sections were then washed and incubated with a mouse CD45 monoclonal IgG (Santa Cruz Biotechnology Inc., Europe) for detection of CD45 on all leukocytes of rabbit origin, then detection of the primary antibodies with Alexa Fluor 488 Goat antimouse IgG (H+C) secondary antibodies 1∶100 dilution (Invitrogen Molecular Probes, USA).

For the *in vivo* drug release study, microfilms were retrieved on Day 28 (n = 11). The retrieved samples were rinsed with de-ionized water and dried in the 37°C vacuum oven for a week. Subsequently, the dried samples were dissolved in ACN and the released amounts were quantified using the HPLC. The amounts of PA released were determined by calculating the difference in the initial drug loaded and the residual drug detected. The cumulative percentage of drug released was also derived accordingly.

### Statistical Analysis

The main outcome measures were the clinical scores for 1) iris vessels, 2) anterior chamber cells, 3) anterior chamber flare, 4) vitreous haze and the combined inflammatory score, defined as the sum of the scores for anterior chamber cells and flare. Statistical Package for the Social Sciences version 17.0 (SPSS Inc., Chicago, IL) was used to analyze the data. Ordinal variables were described with medians and analyzed using Mann Whitney U test for independent samples. All p-values are 2 sided with appropriate significance of p<0.05.

## Results

### Prednisolone acetate release from microfilm

**Figure 3:**
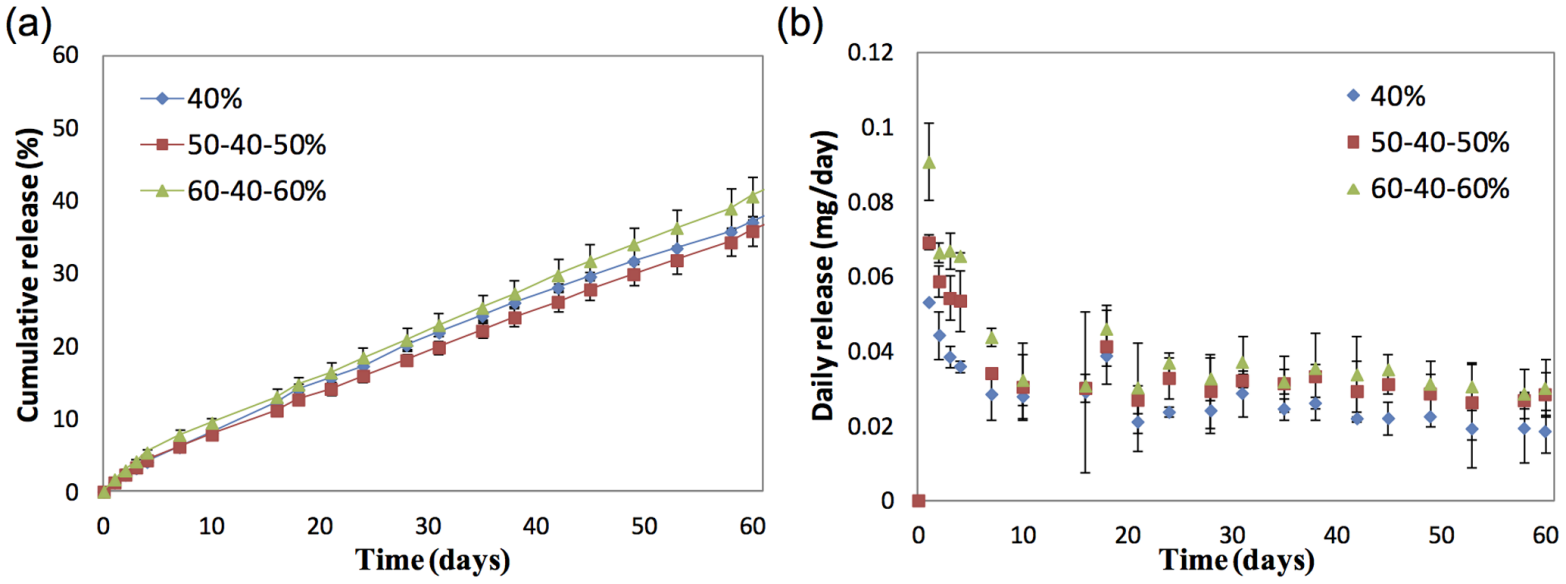
In vitro and In vivo release profiles depicting (a) cumulative release of prednisolone acetate (%) and (b) daily release of prednisolone acetate (mg/day) amount from three formulations of poly(d-, l-lactide-co-ε-caprolactone) films loaded with prednisolone acetate.

**Figure 3a** depicts the cumulative release profile of PA from all three formulations of microfilms over 60 days *in vitro*. All three formulations, regardless of single layer or tri-layer formulations showed an initial burst release followed by steady sustained release of PA. On Day 60, the 40%, 50–40–50% and 60–40–60% loaded microfilms released approximately 37%, 36% and 40% of the initial drug loading, respectively. **Figure 3b** demonstrates the daily release rates of PA from all three formulations of microfilms over 60 days *in vitro*. For the first 4 days, the 60–40–60% formulation displayed highest average release amounts of 0.072 mg/ day, while the 40% and 50–40–50% formulations achieved 0.043 mg/ day and 0.059 mg/ day respectively. The 60–40–60% formulation continued to show relatively higher release amounts throughout the 60 days. For the drug release that followed until day 60, the 40%, 50–40–50% and 60–40–60% displayed average release amounts of 0.025 mg/ day, 0.031 mg/ day and 0.034 mg/ day respectively. From these observations, it appears that although the cumulative release does not differentiate between the 3 formulations, the drug loading percentage affects the daily release.

**Figure 4:**
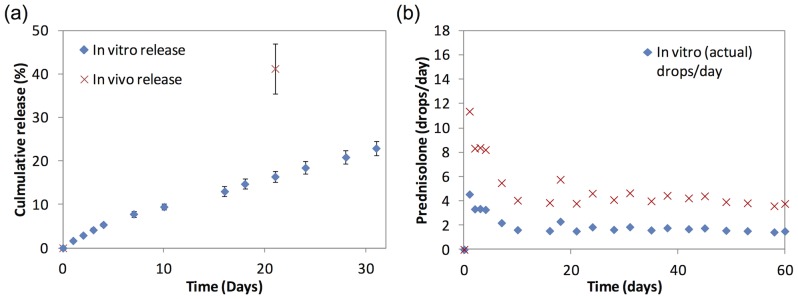
In vitro and in vivo of (a) cumulative release of prednisolone acetate and (b) daily estimated number of prednisolone acetate 1% eye drops per day, from the 60–40–60% prednisolone acetate loaded poly(d-, l-lactide-co-ε-caprolactone) films.

**Figure 4a** plots the *in vitro* and an estimate of *in vivo* cumulative release of the 60–40–60% PA-loaded PLC microfilm formulation. The *in vivo* release was obtained by measuring the residual PA in the microfilms retrieved from the eyes after the animals were sacrificed. At 3 weeks, approximately 16% of PA was released in the *in vitro* study compared to 41% of PA released *in vivo*. **Figure 4b** demonstrates the estimated equivalent number of PA eye drops per day over time. When we correlate the *in vitro* and *in vivo* release, the *in vivo* release rate was calculated to be approximately 2.5 times faster than the *in vitro* release. In clinical practice, prednisolone acetate 1% ophthalmic suspension is used topically. From the estimated rate of release *in vivo*, a corresponding dose to approximately 11.3 drops/day was observed in the first day and 8.3 drops/ day was observed between Days 2 to 4. Subsequently, an estimation of a dose of approximately 4.3 drops per/ day was observed to Day 60.

### Inflammatory scores

**Figure 5:**
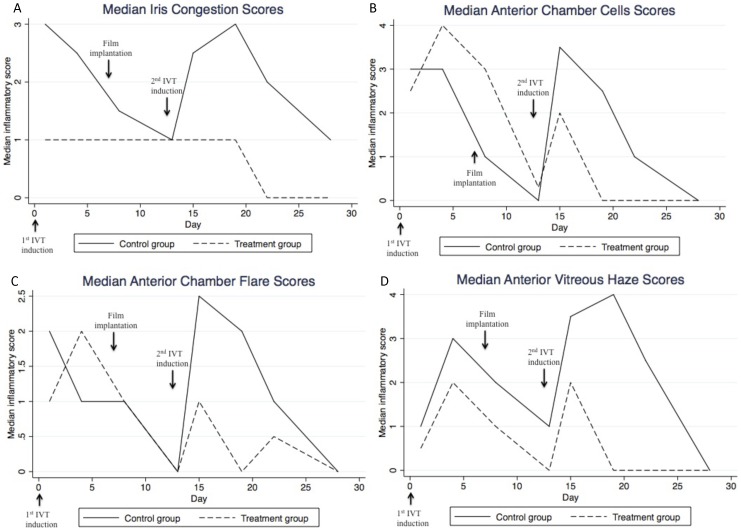
Median inflammatory scores for iris congestion (a), anterior chamber cells (b), anterior chamber flare (c), and vitreous haze (d) - comparing control group and treatment group.

**Figure 6:**
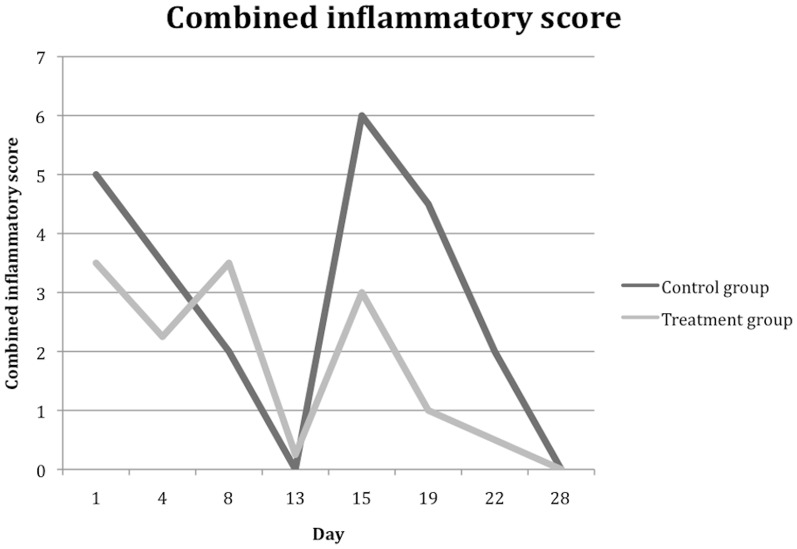
Median combined inflammatory score, as compared between control group and treatment group.

**Table 2** shows the overall inflammatory scores and median scores for iris vessels, anterior chamber cells, anterior chamber flare and vitreous haze during the study period. Just before the second intravitreal induction that simulates a recurrence of uveitis (Day 13), there was no significant baseline difference in the overall inflammatory score (control group: 0 versus treatment group: 0.25, P = 0.96). Following the induction of recurrent uveitis, an increase in scores for all parameters was observed. However, this increase was significantly reduced in the PA-loaded microfilm implanted eyes for all parameters on Day 15 (1 day after the second intravitreal induction, P = 0.04) and Day 19 (5 days after the second intravitreal induction, P<0.001). The difference in iris vessel score remained significantly less in the treatment arm on Day 22. Anterior chamber cells and vitreous haze scores were similarly less in the PA-loaded microfilm implanted eyes on Day 22 but not significantly different by Day 28. Anterior chamber flare was not significantly different between the 2 groups by Day 22. **Figures 5a–d** show the median scores for each parameter in the placebo and treatment arms respectively. The median combined inflammatory scores (**Figure 6**) were significantly lower in the treatment arm on Days 15, 19 and 22 (1, 5 and 8 days after the second intravitreal induction, P = 0.04).

**Table 2:** Median and combined inflammatory scores of control and prednisolone implant groups over study period.

Day	Iris congestion			Anterior chamber cells			Anterior chamber flare			Vitreous haze			Intraocular pressure			Combined inflammatory score			Remarks
	A*	B†	P‡	A*	B†	P‡	A*	B†	P‡	A*	B†	P‡	A*	B†	P‡	A*	B†	P‡	
0	First intravitreal induction																		
1	3	1	<0.001	3	2.5	1.00	2	1	<0.001	1	0.5	0.48	8	15.5	<0.001	5	3.5	0.16	
4	2.5	1	<0.001	3	4	0.04	1	2	0.04	3	2	0.24	9	10.5	1.00	3.5	2.25	1.00	
7	Implantation of microfilms																		
8	1.5	1	0.96	1	3	1.00	1	1	1.00	2	1	0.72	9	14	0.72	2	3.5	1.00	
13	1	1	1.00	0	0.3	0.96	0	0	1.00	1	0	0.06	8	11.5	0.16	0	0.25	0.96	Actual baseline
14	Second intravitreal induction Simulated recurrence of uveitis																		
15	2.5	1	<0.001	3.5	2	0.08	2.5	1	0.06	3.5	2	0.02	7.5	11.5	0.08	6	3	0.04	
19	3	1	<0.001	2.5	0	0.08	2	0	0.008	4	0	<0.001	8	10.5	1.00	4.5	1	<0.001	
22	2	0	0.02	1	0	0.04	1	0.5	0.96	2.5	0	<0.001	7	16.5	<0.001	2	0.50	0.04	
28	1	0	0.24	0	0	1.00	0	0	1.00	0	0	1.00	7.5	13.5	0.60	0	0	1.00	

*A: Control group with blank microfilm implanted eyes.

† B: Treatment group with PA-microfilm implanted eyes.

‡ P values from Mann Whitney U test, comparing control and treatment group at each time point, adjusted with Bonferroni correction.

### Histopathology

**Figure 7:**
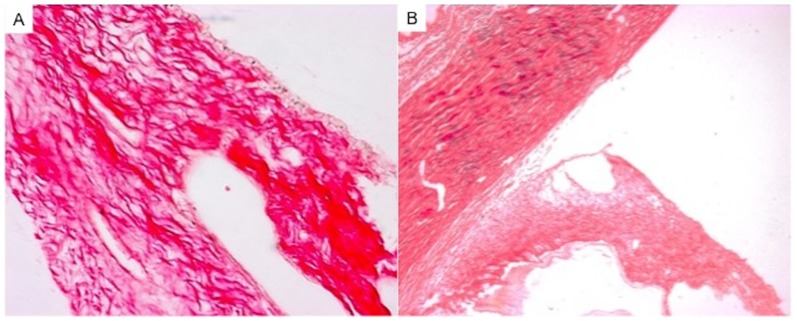
Histology images of the iris and ciliary body stained with Picosirius red. (A) Eye implanted with blank microfilm. (B) Eye implanted with prednisolone acetate loaded microfilm.

**Figure 8:**
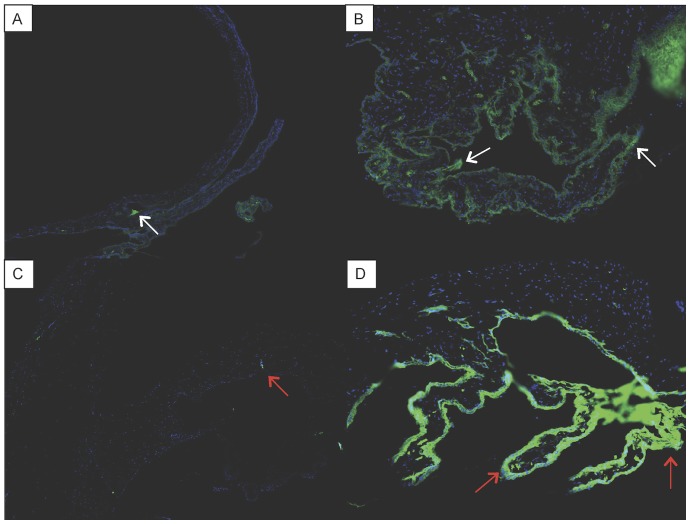
Histology images of the iris and ciliary body with immunohistochemistry stain. (A and C) Eye implanted with prednisolone acetate loaded microfilm. (B and D) Eye implanted with blank microfilm. White arrows: Positive stain for CD45+ leukocytes. Red arrows: Positive stain for CD4+ T cells.

Eyes implanted with the blank microfilm demonstrated a greater intensity in Sirius red staining in the iris and ciliary body than eyes implanted with PA-loaded microfilms (**Figure 7a–b**), indicating the presence of a more significant inflammatory response in the control group. Immunohistochemistry results revealed minimal infiltration of CD45+ leukocytes and CD 4+ T cells in the iris and ciliary body of eyes implanted with PA-loaded microfilms compared to eyes implanted with blank microfilm (mean number of cells from 10 immunohistochemistry stained slides:

Control group  = 14.36±7.57 versus PA-loaded microfilm implant treatment group: 5.66±3.59; P = 0.004 - **Figure 8a–d**).

## Discussion

Visual impairment caused by recurrent uveitis or prolonged ocular inflammation may be prevented if diagnosed and treated adequately[14]. However, current routes for administering corticosteroids to the eyes to treat chronic uveitis have several disadvantages [15]. For example, the current mainstay of topical administration faces the problems of patient complicance and poor intravitreal penetration. Although both periocular or intravitreal injections can provide higher doses of corticosteroids locally, frequent injections may increase the risk of sight threatening complications such as endophthalmitis. Thus, a new treatment option to deliver local therapeutic levels of corticosteroids over a sustained treatment period would be a welcome addition to the clinician's armamentarium against ocular inflammation. Thus, we sought to develop a sustained release PA-loaded microfilm that could be safely implanted in the subconjunctival space, demonstrating its efficacy in a recurrent uveitis animal model in this pilot study.[11] From our *in vivo* studies, an estimated sustained release of 0.12 mg/day PA was achieved over a period of 60 days. This is comparable to the dosing provided by a single drop of Predforte (prednisolone acetate ophthalmic suspension, USP) 1% every 2–3 hours used for treating acute uveitis. Although both the treatment and control groups demonstrated 2 episodes of ocular inflammation which mimics the normal cycle seen with ‘recurrent uveitis’, the treatment group demonstrated a significant attenuation in the magnitude of inflammatory response compared to the control group (P = 0.005).

Currently, topical application of corticosteroids remains the most common route for treating ocular inflammation and uveitis[16]. Thus, our initial *in vitro* and *in vivo* studies sought to demonstrate that the subconjunctival implant provided levels of PA-release similar to therapeutic levels from topical application. First, our *in vitro* studies found that all formulations demonstrated a favourable an initial burst followed by steady subsequent release of PA. However, we found that the 60–40–60% sandwich formulation was the most optimal, releasing 0.065–0.090 mg of PA in the initial release followed by 0.034 mg/day of PA over the period of 60 days. This formulation provides approximately 8 drops per day for the first 4 days followed by approximately 4 drops per day up to 60 days. Similar to our clinical practice, patients with moderate to severe uveitis are prescribed topical corticosteroids such as Predforte (prednisolone acetate ophthalmic suspension, USP) 1% every 1–2 hours for the initial 2–4 days during the awaking hours of the patient; and the dose is subsequently tapered to 4 times a day if ocular inflammation deceases. Thus, this 60–40–60% sandwich formulation was used for the subsequent *in vivo* study, which demonstrated that the PA-loaded microfilms reduced the inflammatory response in the second episode of uveitis. Futhermore, our *in vivo* study confirmed that the implanted microfilms were well tolerated without inciting excessive subconjunctival scarring, consistent with a previous study of similar polymeric microfilms implanted in the subconjunctival space could remain stable for up to 6 months [9].

This pilot study further supports the potential usefulness of surgical implantation of sustained-released drug implants within the subconjunctival space to treat ocular inflammation.[6], [7] The subconjunctival location provides a surgically accessible place for insertion and if necessary, easily accessed for removal. This location also bypasses ocular blood and lymphatic barriers. Moreover, the eventual degradation of the poly (d,l-lactide-co-ε-caprolactone) microfilm to harmless by-products allows for repeated implantations without any further surgery (if removal is not needed). In addition to the formulation that was used in the animal study, we also discovered other formulations that may be useful for eyes with less severe ocular inflammation. In particular, the non-sandwich formulation consisting of 40% PA throughout the entire thickness of the microfilm resulted in a release of approximately 5 drops for the first 4 days followed by 3 drops consistently up to 60 days. This profile might be suitable for use in treatment of mild anterior uveitis, which may be recurrent or chronic. However, the efficacy of different drug release profiles needs to be evaluated in further studies comparing mild and severe anterior uveitis models. We recognize the information derived from this pilot study with small numbers and a short duration of study, due to the limited duration of ocular inflammation in the animal model. However, this animal model is the most appropriate for demonstrating chronic and recurrent ocular inflammation available to us currently. Nonetheless, we believe this preliminary experiment provides the needed evidence to study this PA-loaded implant further for the treatment of ocular inflammation. The potential clinical use may not only be isolated to treating recurrent uveitis,[17] but also be useful for macular edema associated with uveitis,[18] and post-operative ocular inflammation such as after glaucoma filtration surgery, corneal graft and cataract surgery.[19]

In conclusion, in this pilot study we have demonstrated that the use of a sustained releasing PA-loaded microfilm implanted in the subconjunctival is effective in suppressing induced inflammation in uveitis model in rabbits. The implantation of such a system may be able to provide an alternative treatment option to current eyedrops that can deliver a consistent and clinically therapeutic amount of PA to the eye without depending on patient compliance to correctly administer their topical medication.
